# Intergenerational Inheritance of Hepatic Steatosis in a Mouse Model of Childhood Obesity: Potential Involvement of Germ-Line microRNAs

**DOI:** 10.3390/nu15051241

**Published:** 2023-03-01

**Authors:** Francesc Ribas-Aulinas, Sílvia Ribo, Eduard Casas, Marta Mourin-Fernandez, Marta Ramon-Krauel, Ruben Diaz, Carles Lerin, Susana G. Kalko, Tanya Vavouri, Josep C. Jimenez-Chillaron

**Affiliations:** 1 Institut de Recerca Sant Joan de Déu (IRSJD), Esplugues, 08950 Barcelona, Spain; 2 Josep Carreras Leukemia Research Institute (IJC), 08916 Badalona, Spain; 3 Centro de Investigación Biomédica en Red de Diabetes y Enfermedades Metabólicas Asociadas (CIBERDEM), Instituto de Salud Carlos III, 28029 Madrid, Spain; 4 Vall d’Hebron Research Institute (VHIR), 08035 Barcelona, Spain; 5 School of Medicine, University of Barcelona, L’Hospitalet, 08907 Barcelona, Spain

**Keywords:** circadian rhythm, intergenerational epigenetic inheritance, childhood obesity, litter size reduction, DNA methylation, small non-coding RNAs

## Abstract

Childhood obesity increases the risk of developing metabolic syndrome later in life. Moreover, metabolic dysfunction may be inherited into the following generation through non-genomic mechanisms, with epigenetics as a plausible candidate. The pathways involved in the development of metabolic dysfunction across generations in the context of childhood obesity remain largely unexplored. We have developed a mouse model of early adiposity by reducing litter size at birth (small litter group, SL: 4 pups/dam; control group, C: 8 pups/dam). Mice raised in small litters (SL) developed obesity, insulin resistance and hepatic steatosis with aging. Strikingly, the offspring of SL males (SL-F1) also developed hepatic steatosis. Paternal transmission of an environmentally induced phenotype strongly suggests epigenetic inheritance. We analyzed the hepatic transcriptome in C-F1 and SL-F1 mice to identify pathways involved in the development of hepatic steatosis. We found that the circadian rhythm and lipid metabolic process were the ontologies with highest significance in the liver of SL-F1 mice. We explored whether DNA methylation and small non-coding RNAs might be involved in mediating intergenerational effects. Sperm DNA methylation was largely altered in SL mice. However, these changes did not correlate with the hepatic transcriptome. Next, we analyzed small non-coding RNA content in the testes of mice from the parental generation. Two miRNAs (miR-457 and miR-201) appeared differentially expressed in the testes of SL-F0 mice. They are known to be expressed in mature spermatozoa, but not in oocytes nor early embryos, and they may regulate the transcription of lipogenic genes, but not clock genes, in hepatocytes. Hence, they are strong candidates to mediate the inheritance of adult hepatic steatosis in our murine model. In conclusion, litter size reduction leads to intergenerational effects through non-genomic mechanisms. In our model, DNA methylation does not seem to play a role on the circadian rhythm nor lipid genes. However, at least two paternal miRNAs might influence the expression of a few lipid-related genes in the first-generation offspring, F1.

## 1. Introduction

Childhood obesity is a major risk factor for chronic adult diseases, including type-2 diabetes, cardiovascular disease, non-alcoholic fatty liver, or some types of cancer [1,2,3], which collectively shorten lifespan [4]. Furthermore, epidemiological and experimental evidence show that early life nutritional imbalances and childhood obesity may also influence the following generation offspring (reviewed in [5,6,7]). For example, a series of retrospective epidemiological studies (the Överkalix cohort) have shown that grand-paternal excessive nutrient availability during the pre-pubertal growth increases the prevalence of diabetes and diabetes-associated mortality in their grandsons (but not their granddaughters) [6,8]. Conversely, limited food supply during the same developmental period increased longevity in the grandchildren, again in a sex-dependent manner. Sex-specific parental inheritance suggests a mechanism of inheritance involving epigenetic modifications in the sexual chromosomes [9,10]. However, the exact molecular mechanisms are far from being fully elucidated, since no biological material is available in this retrospective historical cohort. The Överkalix cohort is now considered part of a broader scenario that recognizes that paternal health may exert have strong effects in the offspring through various non-genetic mechanisms. It has been named as Paternal Origins of Health and Disease (POHaD) [11,12,13]. Furthermore, it can be argued that interventions aimed to improve paternal health might be potential strategies for improving metabolic offspring health.

We have previously developed a mouse model of early adiposity (i.e., childhood obesity) through litter size reduction [14]. Mice bred in small litters (SL) exhibited rapid neonatal growth and developed obesity as early as by age 15 days [15]. As adults, SL mice displayed several metabolic disturbances, including glucose intolerance, insulin resistance, adult obesity, and hepatic steatosis [15,16]. The earliest significant defect was hepatic insulin resistance that was noticeable by postnatal day 15 (PD15). At the molecular level, hepatic steatosis was due primarily to misalignment of the circadian rhythm, which in turn mediated hepatic steatosis [16,17]. Here, we confirmed that hepatic steatosis was transmitted to the offspring (F1) via the paternal lineage [18]. Thus, here we count on an experimental model in which it is feasible to explore the potential contribution of epigenetic mechanisms in mediating metabolic imbalances across generations.

Epigenetic inheritance through the paternal lineage implies that the nutritionally induced epigenetic marks are transmitted through the gametes (sperm) [19]. It has been widely reported that in humans, obesity, aging or dietary exposures may modify the sperm epigenome [20,21,22,23,24,25]. It is proposed that these modifications might be maintained during the processes of fecundation and embryogenesis and impact on offspring metabolic health [12,26]. However, due to obvious ethical issues, it is really difficult to demonstrate that sperm-harboring epigenetic marks may play a causal role on offspring physiology in humans.

This is easier to address in experimental models (mice and rats), because the males are typically removed from the cage when the female is pregnant. Hence, the male can only contribute to his offspring through the information contained in the spermatozoa [5]. In these experimental procedures, male physiology or behavior does not play a role on offspring phenotypic outcomes. Many laboratories, including ours, have provided compelling data supporting that several environmental challenges, including endocrine-disrupting chemicals, nutrition, pharmacological compounds, and behavioral factors may influence the germline epigenome [27,28,29,30]. Examples of germline epigenetic variation due to nutritional challenges include DNA methylation and small non-coding RNAs, which have been reported in many species, including humans [21,26,31,32,33,34,35,36,37].

Here, we aimed to shed light on the mechanism/pathways that may link paternal early overnutrition-overweight (in SL-F0 mice) with offspring metabolic phenotypes, primarily hepatic steatosis. Specifically, here, we conducted an unbiased transcriptomic analysis in the livers of the C-F1 and SL-F1 mice and found that the circadian rhythm ranked among the most significantly deregulated Gene Ontology term. In turn, the circadian rhythm might underlie altered lipid metabolism in SL-F1 mice. Next, we explored whether the germline epigenome (DNA methylation and small non-coding RNAs) might be involved in the inheritance of hepatic clock genes.

## 2. Materials and Methods

### 2.1. Animal Care and Experimental Design

**Figure 1:**
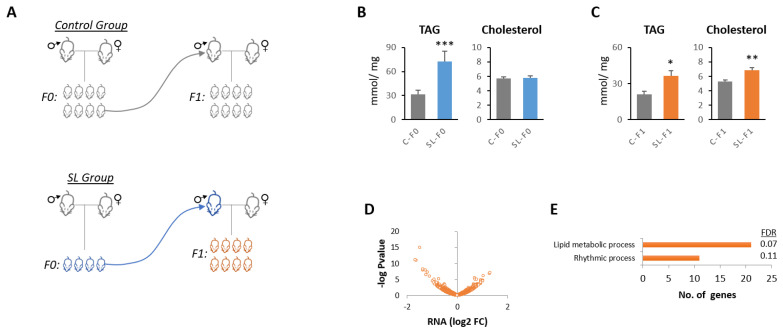
Ancestral overfeeding during early life influences the expression of clock genes in the liver of the next-generation offspring. (**A**) Mouse model of early overfeeding through liter size reduction (SL). In the following generation offspring, F1, litter size was adjusted to 8 mice on each group to normalize postnatal metabolism among groups. (**B**,**C**) Hepatic triglyceride and cholesterol content in the liver of 6-month-old C-F0, SL-F0, C-F1 and SL-F1 male mice. Data represent the means ± standard error. (**D**) Volcano plot including the genes that displayed significant expression between C-F1 and SL-F1 mice. (**E**) GO terms with lowest False Discovery Rates. A.U.: Arbitrary Units; FDR: False Discovery Rate. * *p* < 0.05; ** *p* < 0.01; *** *p* < 0.001, Student’s *t* test. N ≥ 6 in panels (**B**,**C**).

Protocols were approved by the Universitat de Barcelona Animal Care and Use Committee, as previously described [18]. Eight-week-old virgin females were mated with not sibling males (ICR-CD1, Envigo Laboratories, Sant Feliu de Codines, Spain). Upon pregnancy, females were housed individually with ad libitum access to standard chow (2014 Tekland Global, Envigo Laboratories, Spain). Mice were maintained under constant temperature (21–23 °C), humidity (55 ± 10%) and dark–light cycles (12 h/12 h). After delivery, litter size was adjusted to 8 pups (control group, C) or 4 pups per dam (small litter group, SL). Both C and SL offspring are designated as the parental generation, F0 (Figure 1A). F0 pups were nursed freely and weaned at 3 weeks onto standard chow. C-F0 and SL-F0 males were mated at age 3 months with non-sibling external control females to generate the first generation-offspring, F1 (Figure 1A). At birth, all litters were adjusted to 8 pups per dam to normalize early neonatal nutrition and growth.

Mice were euthanized, after 12 h fasting, via CO_2_ inhalation. The liver was weighted, rapidly frozen in liquid nitrogen, and stored at −80 °C for further analyses. In this study, we only included males because, as previously reported, SL females were protected against hepatic steatosis and hepatic insulin resistance [15].

### 2.2. TAG and Cholesterol Determination

Hepatic lipid and cholesterol content were determined from frozen tissue as previously described [16].

### 2.3. Tissue Culture and Incubations with AZA

The murine hepatocyte Hepa-1c cell line was cultured as previously described [34]. Briefly, Hepa1c cells were maintained under standard growth conditions (DMEM, 10% fetal bovine serum). The 60–70% confluent cells were treated with increasing concentrations 5-AZA (Merck, Madrid, Spain) or vehicle for 48 h.

### 2.4. Sperm Isolation

Sperm was isolated from 4-month-old mice as previously described [34]. Briefly, the reproductive tract was retrieved from the mouse. The epididymal conduct of both sides was punctured with a needle, and sperm was isolated by gently shaking the epididymis. The sperm was collected in a culture dish containing warmed PBS solution. Purity of the sperm was confirmed by microscopy.

### 2.5. DNA and RNA Extraction

Genomic DNA from tissues was extracted using the Wizard^®^ Genomic DNA Purification Systems Kit (Promega Biotech Ibérica S.L., Madrid, Spain). Sperm DNA was isolated by using the DNeasy Blood & Tissue Kit (Izasa-Qiagen, Barcelona, Spain). Total RNA was isolated by using TriReagent (Sigma-Aldrich, Madrid, Spain) according to the manufacturers’ protocol.

### 2.6. Affymetrix Microarrays

Microarray hybridization and analysis have been performed as previously described, using GeneChip^®^ Affymetrix Mouse 430 2.0 whole genome arrays (Thermo Fisher Scientific, Sant Cugat del Vallès, Spain) [34]. Briefly, 3 microarrays were hybridized for each group (C-F1, SL-F1). Each array contained the pooled RNA from three independent mice. Expression values were summarized after background correction and normalization steps using the RMA methodology [38]. Differential expression analysis was performed by the non-parametric approach Rank Prod [39]. Oligonucleotides presenting changes between groups with q-values lower than 0.1 were considered significant. The tool David [40] was used for the calculation of the functional clustering enrichment statistical analysis of the Gene Ontology Terms and Kegg Pathways databases considering the list of significant genes. The data have been deposited at the GEO, accession number GSE55304.

### 2.7. Agilent DNA Methylation Microarrays

For CpG island microarray, genomic DNA from C and SL mice was enriched for the unmethylated fraction. Briefly, 500 ng genomic DNA was divided onto two fractions. One of them (250 ng) was subjected to immunoprecipitation. The DNA libraries of immuno-precipitated and non-precipitated samples were labeled (Cy3 and Cy5, respectively) and hybridized onto Agilent 105K Mouse CpG Island microarrays (ID 015279). Before microarray data analysis, outliers and low signal intensity within 2.6 standard deviations of background were removed (Feature Extraction software v.10.7, Agilent Technologies, Santa Clara, CA, USA). Likewise, background was normalized by using the normexp method setting the offset at 10. After normalization, sample DNA methylation and detection were performed by using the Agilent Genomic Workbench, which provides the algorithms for methylation detection. Here, for measuring the degree of enrichment (or de-enrichment) by the methylation-enrichment step, we used the LogOdds score algorithm [41]. Briefly, we compared the overall distribution of enrichment for probes to calculate the probability that the CpG island was methylated or unmethylated (adjusted *p*-value < 0.05). This is expressed as the log of the odds ratio (probability of methylated/probability of unmethylated; LogOdds). Under this condition, a LogOdds score of 0 is equally likely to be methylated/unmethylated. In contrast, large absolute values are increasingly likely to be methylated or unmethylated (positive values or negative values, respectively).

Microarray hybridization and bioinformatics analysis were performed at Bioarray S.L. (Elche, Alicante, Spain).

### 2.8. Small RNA Sequencing and Analysis

Testis RNA samples from 17 different male mice were used for small RNA sequencing. Samples were prepared in two different batches. One of the two batches contained mice from different litters to reduce the confounding effect of genetic relatedness on transcript profiles. All samples had an RNA integrity number of at least 8.0. Libraries were prepared using the TruSeq small RNA preparation protocol with size selection using Pippin prep and sequenced on Illumina Hiseq2500.

The sequencing adaptor (TGGAATTCTCGGGTGCCAAGGAACTCCAGTCAC) was removed using cutadapt version 2.10 [42] requiring a minimum adaptor match of 9 nt (–O 9) and keeping only reads of a minimum read length of 19 nt (-m 19) and a maximum read length of 36 nt (-M 36). Reads were then filtered with the FASTX toolkit 0.0.14 (http://hannonlab.cshl.edu/fastx_toolkit, accessed on 1 January 2022, VBNVNBV) with a minimum quality score of 30 over at least 90% of the read length. Small RNA reads were mapped against the mouse genome (GRCm38) using bowtie version 1.2.3 [43], reporting all best scoring alignments (options–a–best–strata), allowing up to one mismatch (–v 1). We used feature Counts [44] to count reads mapping to tRNAs, microRNAs and known PIWI-interacting RNA loci, requiring a minimum 18 nt overlap between the small RNA and the genomic annotation feature. Multi-mapping reads were counted as a fraction of the number of times they map to the genome. For tRNAs and miRNAs, we counted only reads mapping sense to the genome annotation feature. For piRNAs, we counted reads mapping on both strands. The coordinates of miRNAs were retrieved from miRBase release 22.1. The coordinates of tRNA genes, predicted by tRNAscan-SE [45], were retrieved from the UCSC Genome Browser. The coordinates of loci-producing mouse piRNAs were retrieved from [46]. Differential expression was analyzed using DESeq2 version 1.34 [47] and R version 4.1.2. Differential expression was analyzed by controlling for batch when data sequenced in different batches were used.

### 2.9. Real-Time Quantitative PCR (qPCR)

Total RNA was isolated from frozen tissue (Trizol^®^, Merck, Madrid, Spain) and used for cDNA synthesis (Promega, Barcelona, Spain). Transcript levels were quantified by qPCR using the SYBR Green PCR Master Mix (Promega, Spain). Results were normalized to *b-Actin* and subsequently median-normalized to arbitrary units (A.U.) 1 in the control group. The list of primers is detailed in Table S1.

### 2.10. Statistical Analysis

Results are expressed as mean ± SEM. Statistical analyses were performed using a two-tailed *t* test or a one-way ANOVA as indicated (IBM SPSS Statistics 19, Madrid, Spain). A * *p*-value < 0.05 and *** *p*-value < 0.001 was considered significant.

### 2.11. Data and Resource Availability

The Affymetrix microarray datasets have been deposited at the GEO with accession number GSE55304, and the small RNA sequencing data have been deposited at the GEO with accession number GSE215030 (Accession numbers GSM6620144, GSM6620145, GSM6620150, GSM6620151, GSM6620155, GSM6620159, GSM6620162, GSM6620165, GSM6620166, GSM6620169, GSM6620176).

## 3. Results

### 3.1. Paternal Overnutrition Induced Hepatic Steatosis in the First-Generation Offspring

We have developed a mouse model of early adiposity (i.e., childhood obesity) and long-term metabolic dysfunction through litter size reduction at birth (Figure 1A) [14,15]. Mice reared in small litters (SL-F0) developed hepatic steatosis (increased hepatic triglyceride content) as adults (Figure 1B). The offspring of SL mice (SL-F1, from now onward) also developed glucose intolerance and insulin resistance [18]. Furthermore, here, we confirmed that SL-F1 mice also accumulated greater levels of hepatic triglycerides and cholesterol when compared to the controls (Figure 1C).

We next explored potential pathways involved in the development of hepatic steatosis in SL-F1 mice. We analyzed the global gene expression profiling (Affymetrix) and found that 394 genes were significantly deregulated in the liver of SL-F1 mice (Figure 1D, Table S2). Two ontologies appeared nearly significantly deregulated: The Circadian Rhythm (GO: 0007623) and Lipid Metabolic Process (GO:0006629) (Figure 1E).

### 3.2. Litter Size Reduction Altered the Expression of Genes Involved in the Circadian Rhythm and Lipid Metabolism

**Figure 2:**
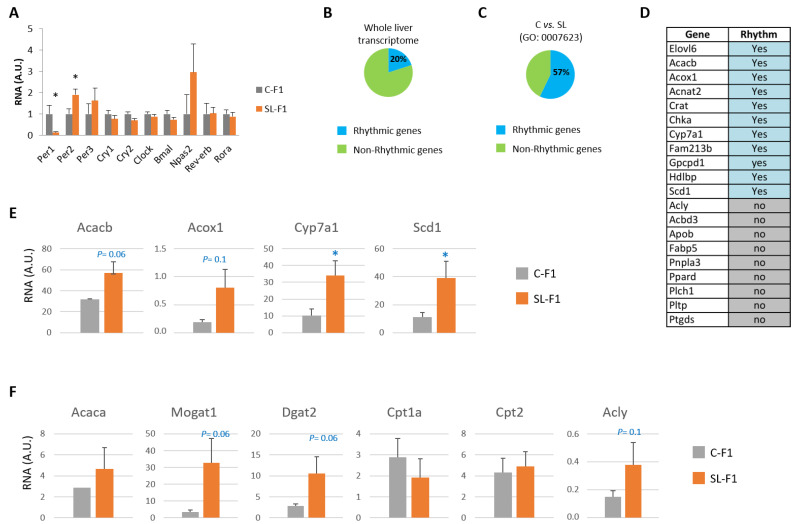
Deregulated circadian rhythm may underlie hepatic lipid metabolic dysfunction. (**A**) Expression (qPCR) of core clock genes and clock-controlled genes in the liver of 5-month-old C-F1 and SL-F1mice. (**B**) Percentage of genes that shows rhythmic behavior in the liver. (**C**) Percentage of differentially expressed genes, included in lipid metabolic process ontology, which display rhythmicity according to the information included in the database CircadiOmics (http://circadiomics.ics.uci.edu; accessed in 1 January 2022). (**D**) List of genes that exhibit rhythmic or non-rhythmic behavior. mRNA levels (qPCR) of rhythmic lipogenic genes (**E**), hepatic lipogenic genes that do not exhibit rhythmicity (**F**), and genes involved in lipid oxidation (**F**), in 4–5-month-old C-F1 and SL-F1 mice. A.U.: Arbitrary Units. * *p* < 0.05, Student’s *t* test. N ≥ 6 in panels (**A**,**E**,**F**).

Strikingly, the previous ontologies appeared already altered in the liver of the progenitors (SL-F0 mice) [16]. In addition, we provided evidence that in SL-F0 mice, the clock genes played a causal role in mediating hepatic steatosis through regulating lipid metabolism. Therefore, here, we explored whether hepatic steatosis in SL-F1 mice might be also attributed, in part, to a similar process. First, we found that some important core clock genes (*Per3*, *Cry1*, *Npas2*) and downstream clock-controlled genes (*Dbp1*, *Nfil3*) appeared in this ontology (Table S3). Next, we confirmed (qPCR) that *Period 1* and *Period 2*, which are involved in regulating lipid metabolism, were deregulated in the liver of adult SL-F1 mice (Figure 2A). Together, these data support that ancestral nutrition in SL-F0 males might program the expression of a few hepatic clock genes in the next-generation offspring, F1. As reported in the F0, it might be possible that the clock genes influence, in part, the expression of lipid-related genes. It is known that 20% of the liver transcriptome exhibits rhythmic behavior (Figure 2B) [48]. In our dataset (Table S4), 57% of the genes included in the ontology (GO: 0006629) displayed rhythmic behavior (Figure 2C,D). This over-representation supports the idea that to some extent, the circadian rhythm might influence hepatic lipid metabolism in SL-F1 mice.

We next validated some of them in the liver of 4–5-month-old mice (Figure 2E). Furthermore, we also confirmed that other lipogenic genes, which do not display cyclic behavior, were also deregulated in the liver of SL-F1 mice (*Mogat1*, *Dgat2*, *Acly*) (Figure 2F). In contrast, target genes involved in lipid oxidation, such as *Cpt1a*, *Cpt2* were unaltered in SL-F1 mice (Figure 2F). Together, these results support that lipid accumulation in SL-F1 mice might be the result of lipid synthesis, which might be marginally dependent of the circadian clock.

### 3.3. DNA Methylation Did Not Correlate with the Expression of Clock and Lipid-Related Genes

**Figure 3:**
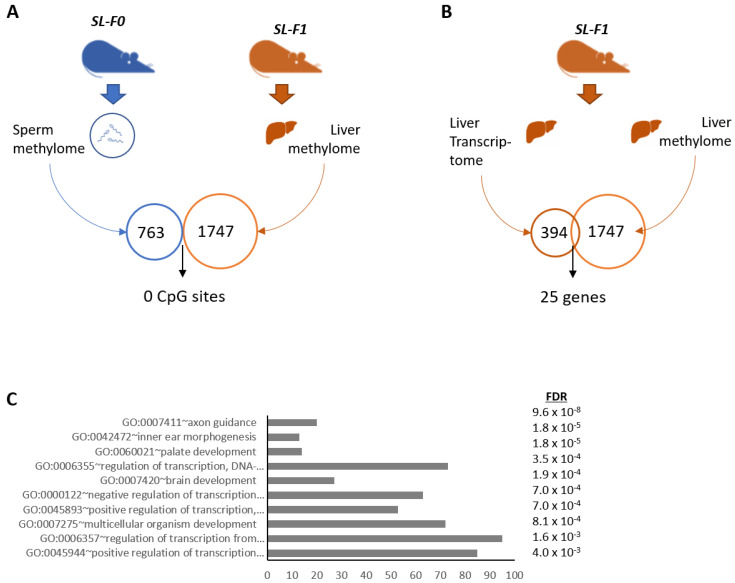
Sperm DNA methylation does not likely regulate gene expression profile in the liver of SL-F1 mice. (**A**) Differentially methylated CpG sites (763) in the sperm samples of C-F0 and SL-F0 and its correlation with 1747 CpG sites differentially methylated in the liver of of C-F1 and SL-F1 mice. (**B**) Correlation between liver transcriptome and liver methylome in F1 mice. Transcriptome and methylome correspond to the genes and CpG sites that appeared differentially expressed between C-F1 and SL-F1 mice. (**C**) GO terms significantly altered in the sperm methylome of SL-F0 mice. FDR: False Discovery Rate.

The inheritance of nutritionally acquired phenotypes is likely attributable to non-genetic mechanisms, with DNA methylation and/or non-coding RNAs as plausible candidates [27,49]. Therefore, here, we first analyzed the DNA methylation profile in the sperm of male mice. We found that 763 CpG sites appeared differentially methylated in the sperm of SL-F0 male mice (Figure 3A; Table S5). These data agrees with previous reports suggesting that obesity and diet may modify germ-line DNA methylation. We next analyzed DNA methylation in the liver of the offspring (C-F1 and SL-F1). We found that 1747 CpG sites were differentially methylated in the liver of SL-F1 when compared to the controls (Figure 3A; Table S6). However, none of the differentially methylated CpG sites was present in both tissues (Figure 3A). These data suggest that nutritionally induced changes in methylation are not transmitted to the offspring.

Furthermore, we tested any associations between the hepatic transcriptome and hepatic methylome (Figure 3B, Table S7). Twenty genes out of 394 appeared to be potentially deregulated in association with changes in DNA methylation. These data suggest that only 6% of our differentially expressed transcriptome is potentially under the control of DNA methylation. In summary, our results indicate that early nutrition may change patterns of sperm DNA methylation. We have no evidence that they are transmitted onto the offspring (at least into the liver) and, therefore, influence the hepatic transcriptome. In support, the ontologies associated to the sperm methyl marks included primarily GOs associated to regulation of gene expression and development (Figure 3C), which did not match the ontologies associated to the transcriptome (Figure 1D). Together, DNA methylation does not seem to play a major role in regulating the hepatic transcriptome in our model.

### 3.4. Two miRNAs Were Differentially Expressed in the Testes of SL-F0 Mice and Might Be Linked to the Hepatic Lipid Metabolism in the Offspring

**Figure 4:**
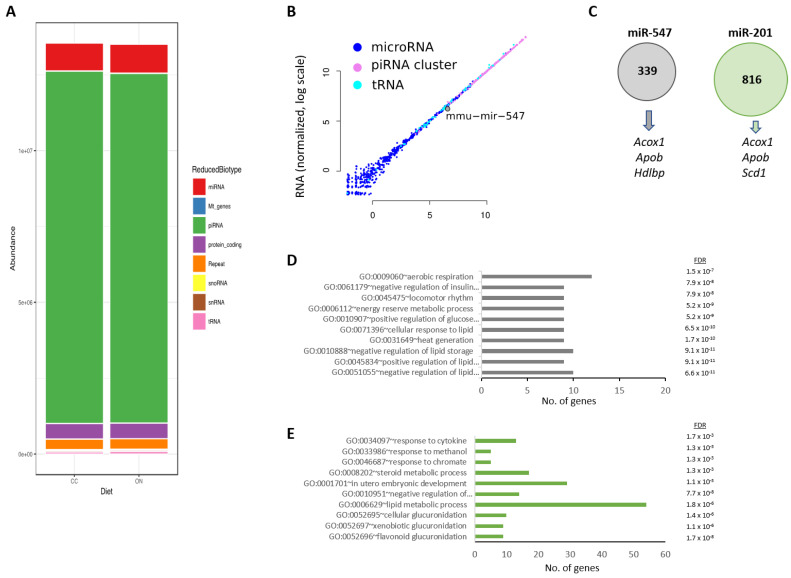
Small non-coding RNAs do not likely regulate clock gene expression in the liver of SL-F1 mice. (**A**) Representative plot of the different type of sncRNAs in the whole testes of C-F0 and Sl-F0 males. Abundance differences of microRNAs (miRNA), tRNA fragments (tRFs) and piRNAs (specifically small RNAs mapping to piRNA-producing loci) between C and SL-F0 animals and their offspring were tested. (**B**) Plot representing the different abundance of all classes of small RNAs between C-F0 and SL-F0 mice groups. (**C**) miR-547 and miR-201 targetome in hepatocytes. The number of mRNA targets for both miRNAs is included in the circles. Targets that appeared also in our liver transcription profile are included. (**D**) Most significant ontologies associated with miR-547 targetome, and (**E**) ontologies associated to miR-201 tartgetome. FDR: False Discovery Rate.

Some intergenerationally transmissible phenotypes have been linked to an altered abundance of sperm-borne small non-coding RNAs [50,51]. We therefore tested the association between small RNAs expressed in the male germline with metabolic dysfunction in the offspring. We sequenced small RNAs from whole testes and searched for differences in the abundance of microRNAs (miRNA), tRNA fragments (TRFs) and piRNAs (specifically mapping to piRNA-producing loci) between C-F0 and SL-F0 mice. First, and as previously published, we confirmed that piRNAs were the most abundant species of sncRNAs in the testes of either group (Figure 4A) [52].

**Figure 5:**
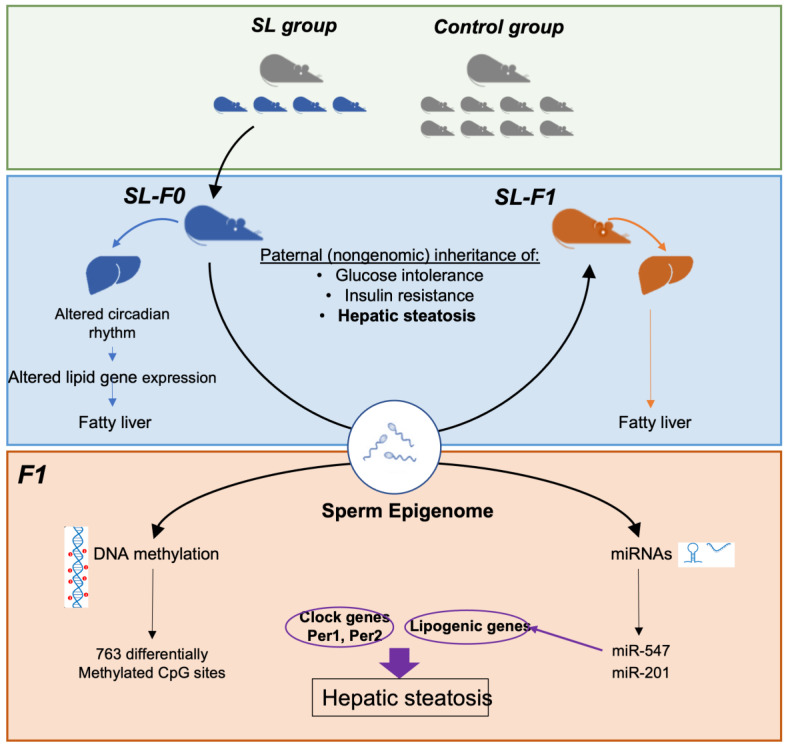
Summary scheme. The (**upper**) panel depicts the mouse model of early adiposity induced by litter size reduction at birth. We reduced litter size to 4 pups/dam in the small litter group (SL) and maintained 8 pups/dam in the control (C) group. The (**medium**) panel summarizes the physiology of adult SL-F0 (blue) and SL-F1 (orange) male mice. SL-F0 mice developed obesity, glucose intolerance, insulin resistance, and hepatic steatosis. Likewise, SL-F1 mice also developed glucose intolerance, insulin resistance and steatosis but not obesity. In SL-F0 mice, steatosis was attributed to a misexpression of clock genes that, in turn, modified the expression of lipid-related genes. Here, we describe that the expression of core clock genes and some lipid-related genes is also deregulated in the liver of SL-F1 mice. However, we do not have compelling evidence that the circadian rhythm is also involved in the development of hepatic steatosis in SL-F1 mice. The intergenerational transmission of altered phenotypes might be due, in part, to germline epigenetic modifications. In the (**lower**) panel, we represent the potential contribution of DNA methylation and small non-coding RNAs. Here, we did not find evidence to support that DNA methylation might be involved in the inheritance of metabolic phenotypes. In contrast, two miRNAs might play a role in the paternal inheritance of hepatic steatosis in our model.

The abundance of all classes of small RNAs was very similar between groups (Figure 4B, Table S8). Only two miRNAs appeared differentially expressed between groups (mmu-miR-547, mmu-miR-201; adj *p*-values 0.01 and 0.08, respectively). Both miRNAs map together within the same chromosome and regulate one each other [53]. In addition, it is described that both are expressed in mature spermatozoa but not in oocytes [54]. Finally, both miRNAs are still detectable in the morula stage but not in the blastocyst. These data support the possibility that they might be paternally inherited. The question again was whether these miRNAs might regulate either clock genes or lipid-related genes. We took advantage that the hepatic miRNA targetome has been recently reported [55]. First, we found that the miR-547 may interact with 339 transcripts, whereas miR-201 could regulate the expression of 816 mRNAs in murine hepatocytes (Figure 4C, Table S9). Interestingly, the miR-547-associated most significant ontology included the regulation of lipid biosynthetic process (GO: 0051055, GO: 0045834, GO: 0010888) (Figure 4D). Likewise, most significant ontologies associated to miR-201 included flavonoid (GO:0052696) and the lipid metabolic process (GO: 0006629) (Figure 4E). Prominently, *Acox1*, *Apob* or *Scd1*, which appeared differentially expressed in the transcriptome assay, are potential targets for both miRNAs (Figure 4C). In contrast, neither miR-201 nor miR-457 targeted any clock gene. Together, these data support the possibility that at least two paternally derived miRNAs (miR201, miR547) might be transmitted to the following generation and target genes involved in hepatic lipid homeostasis (Figure 5).

## 4. Discussion

We have previously developed a mouse model of early adiposity through litter size reduction [14]. The model closely portrays the human pathophysiology associated to childhood obesity. First, litter size reduction led to transient neonatal hyperphagia and obesity onset as early as by postnatal day 7 (PD7) [15]. Next, SL-F0 mice developed progressive insulin resistance, glucose intolerance, hyperglycemia, and hepatic steatosis with ageing [15]. Hepatic steatosis in SL-F0 mice was attributed, in part, to the misexpression of clock genes, which, in turn, influenced the transcription of genes involved in lipid homeostasis [16,17] (Figure 5).

Interestingly, some SL-F0 metabolic phenotypes, including glucose intolerance and insulin resistance, were paternally transmitted to the following generation offspring, which is likely through epigenetic mechanisms (Figure 5) [18]. Here, we confirmed that the offspring of SL-F0 mice (SL-F1 mice) also developed hepatic steatosis (i.e., elevated triglyceride content), albeit at a lower degree than in the parental generation. We speculate that progressive weakening of the phenotype is compatible with a process associated to epigenetic inheritance. Conversely, if hepatic steatosis was due to genetic mutations, the phenotype should remain similar in both generations. At the molecular level, unsupervised transcription profiling uncovered that the ontologies with a lower False Discovery Rate were the circadian rhythm (GO: 0007623; FDR = 0.11) and lipid metabolic process (GO: 0006629; FDR = 0.08). Noteworthy, these ontologies also appeared significantly altered in the liver of their progenitors, SL-F0 mice, with higher significance. Again, these data support that the intergenerational transmission of hepatic steatosis might be likely attributed to germ-line-mediated epigenetic modifications.

In SL-F0 mice, the circadian rhythm played a causal role in the development of hepatic steatosis through modulating the expression of lipogenic genes [13]. Specifically, the *Period* and *Cryptochrome* genes, which can regulate lipid metabolism [16,56,57], were highly dysregulated in SL-F0 mice. Therefore, here, we explored whether in SL-F1 mice lipid metabolism might also be under the control of the clock genes (Figure 5). We confirmed, via qPCR, that *Per1* and *Per2*, but not *Per3*, were misexpressed in the liver of SL-F1 mice. Neither *Cry1* nor *Cry2* were altered in the liver of SL-F1 male mice. Therefore, although we could identify some changes in key period genes, the minimal number of changes does not provide compelling evidence to support that, as in SL-F0 mice, the circadian rhythm is causally influencing the expression of lipid-related genes. Hence, here, we propose that in the liver of SL-F1 mice, the dysregulation of lipid metabolism and hepatic steatosis is not likely due to dysfunction of the circadian rhythm. To fully elucidate whether the circadian rhythm might be involved in our model would require recording gene expression and/or TAG hepatic dynamics throughout a 24 h cycle. This issue needs to be evaluated in the future.

Furthermore, we postulate that paternal transmission of the environmentally induced hepatic steatosis is likely attributed to germline epigenetic modifications (Figure 5) [27,28,58]. Among epigenetic marks, DNA methylation and non-coding RNAs have been recognized as plausible carriers of epigenetic information across generations in response to nutritional challenges [31,33,34,59]. Therefore, here, we set to study whether germline DNA methylation and/or small non-coding RNAs (sncRNAs) might be involved in regulating lipid metabolism in the liver of SL-F1 males. To note, here, we focused on the paternal inheritance only for two reasons: Firstly, we had shown that SL-F0 females did not develop hepatic steatosis and insulin resistance [15]. Therefore, we focused on the robust inheritance of hepatic steatosis that occurred through the male lineage. Secondly, intergenerational effects through the male lineage should be mediated, primarily, by epigenetic mechanisms. In contrast, maternally mediated transgenerational effects will be due to a complex interplay between metabolic, behavioral, mitochondrial, and epigenetic modifications [19,27]. Therefore, from a mechanistic perspective, paternal inheritance is simpler than maternal effects and, hence, dissecting the potential molecular mechanisms involved in these processes is far easier in males than females.

Here, we found that 763 CpG sites appeared differentially methylated in the sperm of SL-F0 mice when compared to the controls (Figure 5). These data agree with previous articles demonstrating that nutritional challenges, obesity, or diabetes may influence the sperm methylome [59,60,61,62,63,64,65]. The question is whether these germline nutritionally induced methyl marks are (i) truly inherited and (ii) influence gene expression profile in target organs of the next-generation offspring, F1. In order to address the first question (are sperm methyl-marks inherited?), we determined the DNA methylation profile in the liver of C-F1 and SL-F1 mice. In total, 1747 CpG sites were differentially methylated in the liver of SL-F1 mice. Yet, no CpG sites were differentially methylated in common: sperm-SL-F0 and liver-SL-F1. These data strongly suggest that environmentally-induced sperm methyl marks are not inherited by the following generation. In agreement, several authors questioned whether sperm DNA methylation might be the actual carrier of epigenetic information across generations at least in the context of metabolic diseases [66,67]. In this regard, it is generally acknowledged that nutritional or metabolic challenges induce small changes in sperm DNA methylation, typically between 5 and 10% [67]. Furthermore, in some examples, sperm-worn methyl marks do not re-appear in the tissues of the offspring [31,66,68,69]. Despite these concerns, the truth is that the potential role of germline DNA methylation as a carrier of epigenetic inheritance is a matter of ongoing debate, with some examples in support [34,58] and a few others against [31,66,67]. We argue that part of these discrepancies should be attributed to the combination between the type of environmental challenge (diet, disrupting chemical, open diabetes, etc.), the window of exposure to the challenge (in utero, early lactation period, adulthood), or the species (mice, rat, human). This combinatorial heterogeneity might underlie the differences reported in the literature. Clearly, a systematic review is warranted to obtain a clearer picture regarding the conditions in which DNA methylation might carry information across generations.

Addressing the second question, it is interesting to remark that when we compared the hepatic transcriptome and methylome, only 25 genes appeared in this list. Therefore, only 6% of the misexpressed genes SL-F1 livers can be potentially attributed to changes in DNA methylation. Hence, most of the hepatic transcriptome is likely regulated by other mechanisms. In summary, the previous data suggest that, in our model, germline changes in DNA methylation (SL-F0) are not inherited and do not likely underlie the misexpression of clock and lipid metabolic genes in the liver of SL-F1 mice.

Alternative to DNA methylation, some intergenerationally transmissible phenotypes have been linked to an altered abundance of sperm-borne small non-coding RNAs [50,51,64,70,71]. A few studies have provided proof for a causal role of miRNAs [37,51] and tRNA fragments [35,36] in mediating the inheritance of metabolic dysfunction. We therefore explored the content of sncRNAs in the testes of C-F0 and SL-F0 males. We did not find relevant differences in the three most abundant small RNA species: piRNAs, miRNAs, and TRFs. Yet, two miRNAs showed different statistical abundance between groups: mmu-miR-547 and mmu-miR-201. Sperm miRNAs are one subspecies of sncRNAs that are sensitive to environmental changes, including dietary manipulations and obesity [37,51]. It is intriguing that only two miRNAs, out of several thousands of sncRNAs, appeared differentially expressed in our model. This could be due to several factors. Firstly, miRNAs might be more sensitive to nutritional challenges than the other RNA species. In agreement, other authors have found that obesity–nutrition induces more alterations in the miRNA fraction than in the others [31,37,72,73]. Secondly, the period of exposure (fetal, neonatal, adult life) and intensity may also influence other sncRNAs. For example, high-fat feeding or low-protein feeding for several weeks in adults results in a higher number of changes not only in miRNAs but also on TRFs [31,35,36,37]. Together, the combination between the window of exposure and obesogenic challenge might determine which sncRNAs species are more susceptible of being dysregulated [12]. Additional studies are warranted to fully elucidate these complex interactions.

It is noteworthy that both miRNAs are expressed from the same chromosomic region [53] and coordinately regulate one each other, which might explain their similar statistical significance in our dataset. These two miRNAs are especially attractive in mediating the paternal inheritance of hepatic lipid dysregulation to SL-F1 mice for two reasons:

- First, both miRNAs are expressed in Sertoli cells during postnatal development [53]. Together, they are involved in the maturation of spermatocytes and are prominently expressed in mature spermatozoa but not in oocytes [54]. Hence, they are strong candidates for being carriers of paternal information onto the offspring.
- Second, the hepatic miRNA-targetome has been recently reported [55]. It provides evidence that miR-201 and mir-547 may regulate the expression of lipid-related target genes, including *Acox1*, *Cpt2*, *Apob*, or *Scd1*. Considering that these genes were identified in our hepatic transcriptome, here, we make the case that both microRNAs might be inherited–transmitted from SL-F0 male mice and influence the hepatic lipid transcriptome.

These data support that both miRNA-201 and miR-457 are paternally transmitted and could therefore be potential carriers of information across generations. In support, it has unequivocally shown that miRNAs may influence offspring phenotype. For example, paternal miRNAs from obese male mice have been experimentally transferred into oocyte cytoplasm during fertilization. These miRNAs can modulate gene expression during embryo development [51]. Next, the miRNAs may regulate a vast array of mRNAs. Therefore, a small set of miRNAs might impact on many different cellular functions playing a crucial role in the pathogenesis of metabolic and endocrine dysfunctions, cardiovascular or cancer diseases and infectious illness [50,51].

At this point, we recognize that despite previous evidence supporting the role of miRNAs in mediating the intergenerational inheritance of hepatic steatosis, there are several caveats that need to be considered. Firstly, we recognize that exploring the sncRNA content in testes might be a potential limitation, since the composition of sncRNAs in the sperm, which is the actual carrier of genetic and epigenetic information, and the testes might differ. However, differentiating male germ cells comprise the majority of cells in testes. Therefore, the relative abundance of the different types of small RNAs in testis predominantly reflects their relative abundance in the male germline. In this regard, as previously noted, both miR-201 and miR-547 are abundant in isolated mature mouse sperm cells but not oocytes [54]. In addition, both miRNAs remain abundant in the morula stage but become completely undetectable in blastocysts [54]. These data support that both miRNA-201 and miR-457 are paternally transmitted and could therefore be potential carriers of information across generations.

Secondly, it is unclear how could the miRNAs influence the adult phenotypes, in the F1, if they disappear during development, after the blastocyst stage? It is proposed that paternally inherited RNAs might influence the offspring phenotype through modulating early developmental stages [50]. Upon fertilization, paternally derived RNAs will stay in the cytoplasm of the newly formed egg. As cell division progresses, the set of paternal RNAs will be progressively diluted and replaced by zygotic RNAs. It is postulated that paternally inherited sncRNAs are required for ensuring appropriate early embryo development.

Thirdly, it is well established that miRNAs may regulate a huge number of transcripts (at the translational level) because they can bind mRNAs with some in specificities. In agreement, miR201 and miR547 can potentially modulate the expression of several hundred genes in hepatocytes (i.e., hepatic targetome). However, only a few targets appeared differentially expressed in our transcriptomic dataset. If both miRNAs are intergenerationally inherited and target lipid-related genes, we should expect a greater percentage of genes in the dataset. Here, we speculate that it is likely that part of the effects of these two miRNAs occurs during early embryogenesis rather than in the adult liver and that the hepatic lipid deregulation we observed in SL-F1 mice is secondary to early alterations. Clearly, this hypothesis deserves being investigated in the future. For example, it would be necessary to measure the expression of these miRNAs in the embryos and the adult liver of SL-F1 and C-F1 mice. In addition, in case we detect their presence in the previous tissues, functional assays would be also warranted.

To finish, we recognize that we centered our search on the potential role of germinal DNA methylation and sncRNAs on offspring clock liver transcription profile. Yet, other epigenetic marks, such as histones, could be involved. For example, histone acetylation is essential during chromatin compaction and linked to the epigenetic inheritance during early gametogenesis [74], and circadian rhythm modulation [74,75,76]. Consequently, additional and deeper analyses should be considered in the future to shed light on transgenerational paternal inheritance mechanisms in our model.

To conclude, our multi-omics approach supports that neither DNA methylation nor sncRNAs directly modify or influence the circadian rhythm in the first-generation offspring, F1. Furthermore, we found no evidence that DNA methylation might impact on genes involved in lipid metabolism. We did find, however, that at least two miRNAs might be paternally inherited (miR-201 and miR-547) and influence hepatic lipid metabolism through modifying early embryogenesis and/or through modulating transcription in adult hepatocytes. The elucidation of these two potential mechanisms deserves further investigation.

## 5. Conclusions

We have previously shown that paternal overfeeding during early development (lactation) triggered the inheritance of metabolic disturbances to the following generation offspring, including glucose intolerance, insulin resistance, and hepatic steatosis.

The inheritance of environmentally induced metabolic dysfunction may be attributed to non-genetic mechanisms, namely epigenetic mechanisms.

Hepatic steatosis, in both the parental and first-generation offspring, may be attributed to two ontologies: circadian rhythm and lipid-related genes.

In this study, we find no evidence to support that germline DNA methylation contributes to modulating the hepatic lipid metabolism in SL-F1 male mice.

We found that two germ-line derived micro RNAs might influence hepatic gene expression through either (a) modulating early steps on embryo development or (b) through directly targeting lipid-related genes in adult hepatocytes.

## Data Availability

Data is contained within the article or supplementary material.

## Supplementary Materials

The following are available online at https://www.mdpi.com/article/10.3390/nu15051241/s1, Table S1: List of primers used for qPCR. Table S2: List of genes differentially expressed in the liver of control-F1 and SL-F1 mice (Afffymetrix). Table S3: List of differentially expressed genes, included in the GO0007623: Circadian Rhythm. Table S4: List of differentially expressed genes, included in the GO0006629: Lipid Metabolic Process, including information regarding their rhythmic behavior. Table S5: List of differentially methylated CpG sites (Agilent) in the sperm of control-F0 samples compared to SL-F0 mice. Table S6: List of differentially methylated CpG sites (Agilent) in the liver of control-F1 samples compared to SL-F1 mice. Table S7: List of genes that appeared differentially methylated and differentially expressed in the liver of F1 mice (intersection between Tables S2 and S6). Table S8: List of small non-coding RNAs expressed in the testes of control-F0 and SL-F0 males. Table S9: List of potential targets for the microRNAs miR-201 and miR-547 (miRNA targetome).
