# Supplementary material for: Intergenerational Inheritance of Hepatic Steatosis in a Mouse Model of Childhood Obesity: Potential Involvement of Germ-Line microRNAs
Source: Nutrients. 2023 Mar 1;15(5):1241. doi: 10.3390/nu15051241 (PMC10005268; doi:10.3390/nu15051241)
Supplement: Supplementary file 1 [file nutrients-15-01241-s001.zip › nutrients-2184192-sup1/Table S1.pdf]

Name	5' > 3' Sequence
**Acaca-Fw**	TGGATCCGCTTACAGAGAGACT
**Acaca-Rv**	GCCGGAGCATCTCATTCG
**Acacb-Fw**	CCAGTCTTCCGTGCCTTTGTAC
**Acacb-Rv**	CTCATCCCTCGCTCTGAACG
**Acox1-Fw**	TGTTGATGAAATATGCCCAG
**Acox1-Rv**	CTCGCTTCTCTTGATTTCAG
**b-Actin-Fw**	CCACCGATCCACACAGAGTA
**b-Actin-Rv**	AGTGTGACGTTGACATCCGT
**Acly-Fw**	TGATGGGAGAAGTTGGGAAG
**Acly-Rv**	ATCAGCTCGGGACTCAGAAA
**Bmal1-Fw**	AACCATGTGCGAGTGCAGGCGC
**Bmal-Rv**	GGACTTCGCCTCTACCTGTTCA
**Clock-Fw**	GGAGGGAAAGTGCTCTGTTGTAG
**Clock-Rv**	TTGCTCCACGGGAATCCTT
**Cpt1a-Fw**	CTCAGTGGGAGCGACTCTTCA
**Cpt1a-Rv**	GGCCTCTGTGGTACACGACAA
**Cpt2-Fw**	CAGTGCACAGAAGCCTCTCTTG
**Cpt2-Rv**	CTTCCCAATGCCGTTCTCAA
**Cry1-Fw**	CTGTCCGCCATTGAGTTCTATG
**Cry1-Rv**	CTGGCGTGGAAGTCATCGT
**Cry2-Fw**	GTTTTTCAGGCCCACTCTACCTT
**Cry2-Rv**	AGCCCAGGCCAAGAGGAA
**Cyp7a1-Fw**	GTCCGGATATTCAAGGATGCA
**Cyp7a1-Fw**	AGCAACTAAACAACCTGCCAGTACTA
**Dgat2-Fw1**	CTACTTCCGAGACTACTTTC
**Dgat2-Rv1**	CTGTGCTGAAGTTACAGAAG
**Mogat1-Fw**	GGTGAATGTTCCTGGGTGAG
**Mogat1-Rv**	CTGGTTCTGTTTCCCGTTGT
**Npas2-Fw**	AAGGGATAGAGCAAAGAGAGCCT
**Npas2-Rv**	CATTTTCCGAGTGTTACCAGGG
**Per1-Fw**	TGAAGCAAGACCGGGAGAG
**Per1-Rv**	CACACACGCCGTCACATCA
**Per2-Fw**	GCGGAATCGAATGGGAGAAT
**Per2-Rv**	ATGCTCGCCATCCACAAGA
**Per3-Fw**	AGGTCTTCCCTGGCTTTGTT
**Per3-Rv**	ACAGCCCAATGTCTTCAGGT
**Rev-erb-Fw**	GGTGCGCTTTGCATCGTT
**Rev-erb-Rv**	GGTTGTGCGGCTCAGGAA
**Rora-Fw**	AGCTGCCACATCACCTCTCT
**Rora-Rv**	TCCCCTACTGTTCCTTCACC
**Scd1-Fw**	CTTCACTTCTCTCGTTCATTT
**Scd1-Rv**	ATGCTCCAAGAGGATCTCCAGT
